# A Manual of Dental Anatomy, Human and Comparative

**Published:** 1876-12

**Authors:** 


### BIBLIOGRAPHICAL.

---

*A Manual of Dental Anatomy.*—Human and Comparative. By Charles S. Tomes, M. A. Publishers, Lindsay and Blackiston, Philadelphia, 1876.

The author, who is Lecturer on Dental Anatomy at the London Dental Hospital, in order to supply a want he has long felt for a Manual sufficiently new to embody the advance of late years, and which he could recommend to students as a text book, has prepared this work. The table of contents embodies the Nature and Description of teeth, Maxillary Bones, and Associated Parts, Enamel, Dentine, Cementume, Tooth Pulps, &c. The development of the Teeth, in Fish, in Reptiles, in Mammals, Calcification of the Dental Tissues. The Development of the Jaws and the Eruption and Attachment of the Teeth. The Teeth of Fishes, of Batrachia and Reptiles, of Mammals, Homologies of the Teeth, Milk Dentition, Teeth of Monotremata, Edentata, Cetacea, Ungulata, Sirenia, Hyracoidea, Proboscidea, Rodentia, Carnivora, Insectivora, Chiroptera, Primates, and Marsupialla; comprising some four hundred pages and profusely illustrated with well executed wood engravings, rendering it a useful and instructive work, not only as a text book for dental students, but for the library of the dental practitioner.

The reputation of the author is a sufficient guarantee for the careful maner in which this manual is prepared; and the style in which it appears is creditable to the well known publishers.

